# Generic vs. brand-name pramipexole: effectiveness, safety, and cost comparison in a multicenter retrospective cohort study

**DOI:** 10.3389/fnagi.2026.1873459

**Published:** 2026-06-29

**Authors:** Ke Wang, Xiaoxuan Xing, Zhizhou Wang, Xiaotong Zhang, Jingxuan Li, Ying Zhu, Hao Guo, Changzhi Yu, Lan Zhang, Xianzhe Dong

**Affiliations:** 1 Department of Pharmacy, Xuanwu Hospital of Capital Medical University, Beijing, China; 2 Department of Pharmacy, The Second Hospital of Hebei Medical University, Hebei, Shijiazhuang, China; 3 Department of Pharmacy, The First Affiliated Hospital of Soochow University, Jiangsu, Suzhou, China; 4 Department of Pharmacy, Inner Mongolia Autonomous Region People’s Hospital, Inner Mongolia, Hohhot, China; 5 Department of Pharmacy, The First Affiliated Hospital of Xinjiang Medical University, Xinjiang, Urumqi, China

**Keywords:** cost, effectiveness, generic drug, immediate-release, Parkinson’s disease, pramipexole, safety

## Abstract

**Background:**

Generic pramipexole is widely prescribed for Parkinson’s disease (PD), yet clinical equivalence to the brand-name formulation remains debated. We aimed to compare generic and brand-name pramipexole dihydrochloride tablets in real world.

**Methods:**

We conducted a multicenter retrospective propensity score-matched cohort study. Prescription data of outpatients using either generic or brand-name pramipexole dihydrochloride tablets for PD or Parkinson’s syndrome were extracted at five research hospitals from July 1, 2018, to June 30, 2023. Effectiveness was assessed by changes in levodopa equivalent dose (LED) over time, while safety was evaluated by adverse events (AEs) from enrollment to follow-up completion. Medication costs were also compared between the two groups.

**Results:**

A total of 1,063 patients in the brand-name drug group and 1,287 in the generic drug group were included. A total of 161 AEs occurred in the generic drug group and 182 in the brand-name drug group. There was no significant difference in AE occurrence between the two groups (*P* = 0.898). And no significant difference was found in the change degree of LED level between generic drug and brand-name drug group over time (*H* = 1.514, *P* = 0.824). The daily cost of pramipexole and antiparkinsonian drug, as well as the average prescription cost over 1 year of follow-up in the generic drug group were significantly lower than those in the brand-name drug group (*P* < 0.05).

**Conclusion:**

With comparable efficacy and safety, our results indicate the generic pramipexole should be considered a valuable alternative to the brand-name drug. Future prospective research is needed to confirm these findings in clinical practice.

## 1 Introduction

Parkinson’s disease (PD) is a common neurodegenerative disease characterized by motor symptoms such as bradykinesia, rest tremor, rigidity, and postural instability, as well as various non-motor symptoms (Tolosa et al., 2021). As the disease progresses, individuals with PD may gradually have difficulty in daily activities, lose the ability to care for themselves, and eventually become bedridden (Fabbri et al., 2022; Chen et al., 2022). An estimated 6.1 million people worldwide were living with PD in 2016, and the number continues to rise (Ben-Shlomo et al., 2024; GBD 2016 Parkinson’s Disease Collaborators, 2018; Dorsey et al., 2018). China, in particular, has a large PD population and is experiencing the fastest growth in cases globally (GBD 2016 Parkinson’s Disease Collaborators, 2018). Projections suggest that by 2030, China could have around 5 million individuals suffered from PD, accounting for nearly half of the PD population in the world (Li et al., 2019). This certainly imposes a heavy economic burden on the healthcare system (Boland and Stacy, 2012).

Pharmacological treatments for PD motor symptoms currently include levodopa preparations, dopamine agonists (DAs), monoamine oxidase-B inhibitors, catechol-O-methyltransferase inhibitors, anticholinergics, and amantadine (Armstrong and Okun, 2020; Hayes et al., 2019). Among these, pramipexole, a non-ergolinic DA, remains the most commonly prescribed DA for treatment of PD in China (Yi et al., 2022). It is recommended in various guidelines as an initial monotherapy for early-stage PD patients and as an adjunct to levodopa for those with advanced PD (CSPDMD and PDMD-CMDA, 2020; Grimes et al., 2019; NICE, 2017; Fox et al., 2018).

Pramipexole dihydrochloride was first marketed under the brand name Mirapex by Boehringer Ingelheim Pharma GmbH & Co. KG. It was approved by the Food and Drug Administration (FDA) in 1997 for the treatment of PD and allowed to enter the Chinese market in 2007. Following this, generic versions of pramipexole dihydrochloride were launched in China, significantly reducing drug prices. The approval process for generic drugs is generally simplified and focuses on proving their equivalence to the brand-name drug, which is ensured by conducting bioequivalence (BE) tests between generic and reference products (Charoo, 2020). Since the BE tests are conducted on healthy subjects rather than clinical patients, the differences in physiological and pathological status may lead to clinical inequivalence between generic drugs that pass BE tests and brand-name drugs (Go et al., 2011; Bhushan and Martens, 2024). In addition, variations in absorption characteristics and solubility properties can also lead to differences in effectiveness and safety (Go et al., 2011; Weitzel et al., 2020). Changes in PD symptoms or medication response can sometimes occur due to a transition from the brand-name drug to generic drug (Go et al., 2011). Therefore, doctors and patients still have considerable concerns about the use of generic drugs (Zhang et al., 2021; Zhao et al., 2021). It is necessary to compare the differences between generic and brand-name drugs in clinical practice.

China issued the National Centralized Drug Procurement (NCDP) policy in November 2018, which mandates that certain brand-name drugs and generic drugs passing generic consistency evaluations (GCE), that is, demonstrating pharmaceutical equivalence and BE with the reference drug, be procured through volume-based purchases (Sun et al., 2023). It promoted the substitution of generic drugs and played positive effects on drug price reductions (Yang et al., 2022). The pramipexole dihydrochloride tablet, developed by CSPC Ouyi Pharmaceutical Co., Ltd., was the first generic immediate release pramipexole passing GCE in China and was launched in 2020. It was included in the fourth round of selected drugs for NCDP. However, there is a lack of comparison between the generic and brand-name pramipexole dihydrochloride tablets. Therefore, this study aims to compare the effectiveness, safety and medication costs of generic and brand-name pramipexole dihydrochloride tablets to provide evidence for clinical decision-making.

## 2 Materials and methods

### 2.1 Data source

This retrospective cohort study was registered in the Chinese Clinical Trial Registry on August 2, 2024 (ChiCTR2400087756) and conducted using outpatient prescription records from the electronic information systems of medical institutions. Prescription data of patients treated with either generic or brand-name pramipexole dihydrochloride tablets for PD or Parkinson’s syndrome (ICD-10 code G20 or G21) from July 1, 2018, to June 30, 2023, were extracted from five tertiary hospitals (Xuanwu Hospital of Capital Medical University, the Second Hospital of Hebei Medical University, the First Affiliated Hospital of Soochow University, Inner Mongolia Autonomous Region People’s Hospital, and the First Affiliated Hospital of Xinjiang Medical University). The extracted data included administrative information (such as patient ID, medical insurance type, visit date, and department), demographics (age and gender), disease diagnoses, and medication information (prescription dosage, quantity, and costs of pramipexole as well as any other concomitant medications). All personal identifiers were removed to ensure patient privacy. This study was approved by the Ethics Committee of Beijing Xuanwu Hospital, Capital Medical University [Clinical Scientific Research (2023) No.156].

### 2.2 Study design and population

This study employed a new-user active comparator design. Patients who used brand-name pramipexole between January 1, 2019, and June 30, 2020 (identification period), and those who used generic pramipexole between July 1, 2021, and December 31, 2022 (identification period), were identified for this study. The first prescription date within these periods was considered the index date. The 6 months preceding the index date were defined as the baseline period. Follow-up visits were conducted every 3 months after the index date to collect prescription information. The brand-name drug group was followed up until December 31, 2020, and the generic drug group until June 30, 2023. Patients were further excluded if they: (1) had pramipexole prescription records during the baseline period; (2) lacked pramipexole prescription records within 1 year after the index date, or during the follow-up period (<1 year); (3) used both brand-name and generic pramipexole simultaneously, or used both immediate-release and sustained-release formulations simultaneously; or (4) had incomplete information. For follow-up time points without prescription records, the most recent visit’s prescription data were used for imputation. Any instances of switching medications or discontinuation were considered as loss to follow-up.

### 2.3 Outcome measures

The levodopa equivalent dose (LED) level of PD patients serves as an indirect measure of disease severity and medication intensity (Li et al., 2024; Li et al., 2018). Therefore, the level and speed of LED change over time were used to evaluate the effectiveness of generic versus brand-name pramipexole. We calculated the levodopa equivalent dose (LED) levels for each patient at the index date, and at 3, 6, 9, and 12 months during the follow-up period, following the formula described by Jost et al. (2023).

Safety was measured by the incidence of adverse events (AEs), which were identified through new diagnoses and concomitant medications during the follow-up period, as well as the timing of their occurrence. The diagnostic terms for AEs were identified using the drug label and medical databases (UpToDate, Micromedex). These included: dizziness (ICD-10 code R42.X51), headache (G44.451), insomnia (G47.001), drowsiness (R53.009), sleep disorder (G47.900), hallucination (F19.506), constipation (K59.000), nausea (R11.X02), dyspepsia (K30.X00), hypotension (I95.251), fatigue (R53.X51), weakness (R53.X00X002), and edema (R60.900).

Medication cost outcomes included the daily cost for pramipexole and antiparkinsonian drugs at the index date and each follow-up time point. Additionally, the average prescription cost over the 1-year follow-up period was calculated. Costs were adjusted to 2023 values using the medical care Consumer Price Index (CPI) from the National Bureau of Statistics (China National Bureau of Statistics, 2023) and then converted into US dollars based on the average exchange rate for 2023 (US$1 = 7.05 CNY) (China National Bureau of Statistics, 2024).

Effectiveness and medication cost outcomes were evaluated for patients with follow-up lasting more than 1 year (at least four follow-up records). Safety, on the other hand, was assessed for all included patients.

### 2.4 Statistical analysis

In this study, normally distributed continuous data were expressed as mean ± standard deviation (SD) and compared between groups using the Student’s *t*-test. Non-normally distributed continuous data were expressed as median and interquartile range (IQR) (25th and 75th percentiles) and compared using the non-parametric Mann-Whitney U test. Categorical data were expressed as counts (percentages) and compared using the Chi-square test.

Propensity score matching (PSM) was used to match patients in the two groups at a 1:1 ratio. A propensity score for each patient was estimated using a logistic regression model based on caliper width of 0.02. The dependent variable in this model was the use of generic or brand-name drugs, and potential confounders included age, gender, medical insurance type, number of disease diagnoses, LED levels at the index date, and polypharmacy (use of ≥ 5 medications simultaneously).

To compare differences in LED levels at various follow-up time points, a two-way nonparametric Scheirer-Ray-Hare test was applied, as the data did not follow a normal distribution. This test examined whether LED levels were affected by two factors: follow-up time and drug type (generic or brand-name). Kaplan-Meier survival analysis was performed to calculate the AE-free survival time and the overall incidence rate of AEs, with the log-rank test used to compare differences between the generic and brand-name pramipexole groups. Univariate and multivariate Cox proportional hazards models were employed to estimate hazard ratios (HRs) and their 95% confidence intervals (95% CIs). The Fine-Gray model was used to account for the competing risks posed by treatment switching or discontinuation on the incidence of pramipexole-related AEs.

To assess the robustness of the primary results, we performed sensitivity analyses on the effectiveness outcome, focusing on patients with at least five follow-up records. We conducted sensitivity analyses of safety by including patients who lacked pramipexole prescription records within 1 year after the index date, or during the follow-up period (<1 year).

All statistical analyses were performed using SPSS 26.0 and R 4.3.0. A two-tailed *P*-value of < 0.05 was considered statistically significant.

## 3 Results

### 3.1 Baseline characteristics

**FIGURE 1:**
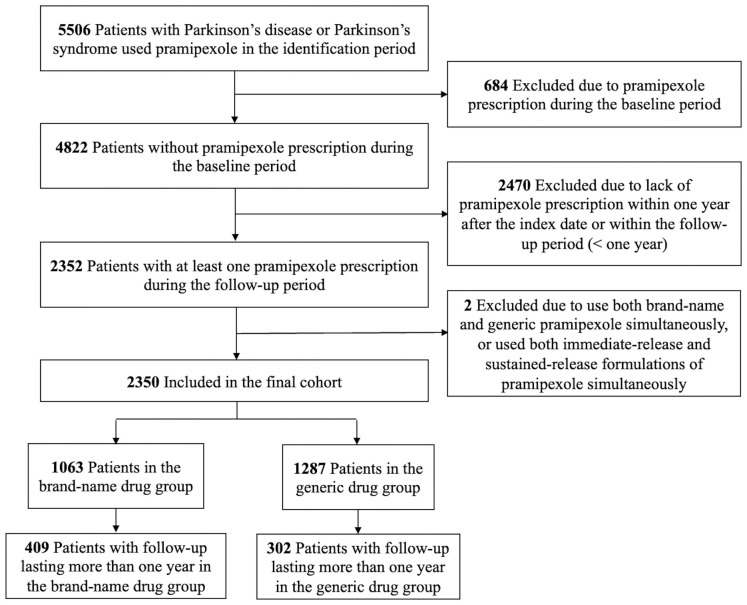
Flowchart of the present study.

A total of 2,350 eligible patients were included in the study cohort (Figure 1). Among them, 1,063 were in the brand-name drug group and 1,287 were in the generic drug group. The baseline characteristics are presented in Table 1. The percentage of patients with health insurance was higher in the brand-name drug group compared to the generic drug group (69.9% vs. 65.2%; *P* = 0.015). After PSM, no significant differences in the baseline characteristics were observed between the brand-name and generic drug groups (all *P* > 0.05). Additionally, histograms showed the distributional overlap of propensity scores became similar in the two groups after matching (Supplementary Figure 1).

**TABLE 1:** Baseline characteristics of patients using brand-name and generic pramipexole.

Characteristics	Unadjusted			Propensity score-matched		
	Brand-name drug (*n* = 1,063)	Generic drug (*n* = 1,287)	*P-*value	Brand-name drug (*n* = 1,063)	Generic drug (*n* = 1,063)	*P-*value
Gender, *n* (%)			0.629			0.896
Male	531 (50.0)	630 (49.0)		531 (50.0)	534 (50.2)	
Female	532 (50.0)	657 (51.0)		532 (50.0)	529 (49.8)	
Age, years; median (IQR)	67.0 (60.0–74.0)	67.0 (61.0–73.0)	0.773	67.0 (60.0–74.0)	67.0 (60.0–73.0)	0.572
Medical insurance type, *n* (%)			0.015			0.161
With health insurance	743 (69.9)	839 (65.2)		743 (69.9)	713 (67.1)	
No health insurance	320 (30.1)	448 (34.8)		320 (30.1)	350 (32.9)	
No. of disease diagnoses, median (IQR)	1 (1–2)	1 (1–2)	0.229	1 (1–2)	1 (1–2)	0.541
LED levels at the index date, mg; median (IQR)	537.5 (150.0–775.0)	675.0 (175.0–825.0)	0.062	537.5 (150.0–775.0)	600.0 (150.0–775.0)	0.431
Patients on polypharmacy, *n* (%)	124 (11.7)	174 (13.5)	0.179	124 (11.7)	129 (12.1)	0.738
Daily dose of pramipexole at the index date, *n* (%)			0.275			0.546
≤ 1.5 mg	994 (93.5)	1,182 (91.8)		994 (93.5)	981 (92.3)	
1.5–2.25 mg	29 (2.7)	48 (3.7)		29 (2.7)	34 (3.2)	
> 2.25 mg	40 (3.8)	57 (4.4)		40 (3.8)	48 (4.5)	

Polypharmacy refers to using of five or more medications simultaneously; IQR, interquartile range; LED, levodopa equivalent dose.

In the brand-name drug group, the patients were followed up for an average of 8.7 months, and 409 patients were followed for more than 1 year. Patients in the generic drug group were followed up for average of 7.3 months, and 302 patients were followed for more than 1 year. The baseline characteristics of these patients are compared in Table 2. The brand-name drug group had a higher proportion of patients with health insurance (83.6% vs. 71.2%; *P* < 0.001), a greater number of disease diagnoses (brand-name drug: median 1, IQR 1–3; generic drug: median 1, IQR 1–2; *P* = 0.049), and fewer patients with the daily dosage of pramipexole higher than 1.5 mg at the index date (4.6% vs. 8.9%; *P* = 0.038). After applying PSM, no significant differences in the baseline characteristics were observed between the brand-name and generic drug groups for patients followed for more than 1 year (all *P* > 0.05). The distributional overlap of propensity scores was similar between the two groups after matching (Supplementary Figure 2).

**TABLE 2:** Baseline characteristics of patients with follow-up lasting more than 1 year using brand-name and generic pramipexole.

Characteristics	Unadjusted			Propensity score-matched		
	Brand-name drug (*n* = 409)	Generic drug (*n* = 302)	*P-*value	Brand-name drug (*n* = 287)	Generic drug (*n* = 287)	*P-*value
Gender, *n* (%)			0.626			0.676
Male	201 (49.1)	154 (51.0)		141 (49.1)	146 (50.9)	
Female	208 (50.9)	148 (49.0)		146 (50.9)	141 (49.1)	
Age, years; median (IQR)	67.0 (60.0–75.0)	68.0 (61.0–74.0)	0.736	66.0 (60.0–74.0)	68.0 (61.0–74.0)	0.245
Medical insurance type, *n* (%)			< 0.001			0.497
With health insurance	342 (83.6)	215 (71.2)		220 (76.7)	213 (74.2)	
No health insurance	67 (16.4)	87 (28.8)		67 (23.3)	74 (25.8)	
No. of disease diagnoses, median (IQR)	1 (1–3)	1 (1–2)	0.049	1 (1–3)	1 (1–2)	0.447
LED levels at the index date, mg; median (IQR)	673.5 (150.0–850.0)	650.0 (150.0–825.0)	0.739	675.0 (150.0–912.5)	650.0 (150.0–825.0)	0.089
Patients on polypharmacy, *n* (%)	53 (13.0)	46 (15.2)	0.387	42 (14.6)	39 (13.6)	0.719
Daily dose of pramipexole at the index date, *n* (%)			0.038			0.212
≤ 1.5 mg	390 (95.4)	275 (91.1)		272 (94.8)	262 (91.3)	
1.5–2.25 mg	6 (1.5)	13 (4.3)		5 (1.7)	11 (3.8)	
> 2.25 mg	13 (3.2)	14 (4.6)		10 (3.5)	14 (4.9)	

Polypharmacy refers to using of five or more medications simultaneously; IQR, interquartile range; LED, levodopa equivalent dose.

### 3.2 Effectiveness outcome

**FIGURE 2:**
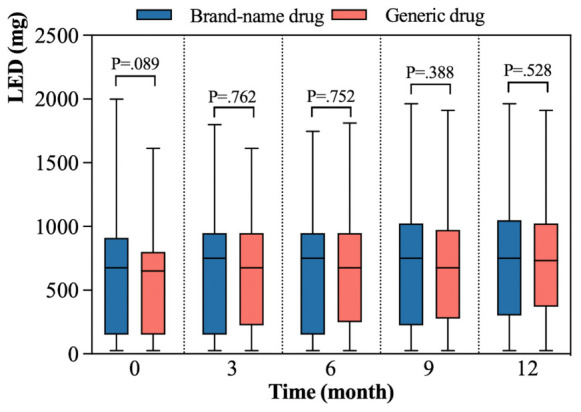
Comparisons of LED levels between brand-name and generic drug groups among patients with follow-up lasting more than 1 year in the matched cohort. The *P*-values for pairwise comparisons at the index date and each follow-up time point were achieved with the non-parametric Mann-Whitney U tests and added for each subgroup in the graph. LED, levodopa equivalent dose.

There were no significant differences in LED levels between the generic and brand-name drug groups at 3, 6, 9, and 12 months during the follow-up period (all *P* > 0.05) (Figure 2). However, significant differences in LED levels were found across different follow-up time points (time: *H* = 42.113, *P* < 0.001), with both groups showing an upward trend in LED levels. There was no significant difference in LED levels between the groups or in the interaction between group and time (group: *H* = 2.791, *P* = 0.095; time × group: *H* = 1.514, *P* = 0.824), indicating that the change in LED levels over time was similar between the generic and brand-name drug groups.

In the sensitivity analyses for effectiveness, 262 patients in the brand-name drug group and 182 patients in the generic drug group had at least five follow-up records. Using PSM, 160 patients from each group were included, with no significant differences in baseline characteristics (all *P* > 0.05; Supplementary Table 1). In this matched cohort, there were also no significant differences in LED levels between the two groups (all *P* > 0.05; Supplementary Table 2). Furthermore, while significant differences in LED levels were found across different follow-up time points (time: *H* = 21.617, *P* < 0.001), no significant differences were noted between the groups or in the interaction between group and time (group: *H* = 3.155, *P* = 0.076; time × group: *H* = 1.722, *P* = 0.787).

### 3.3 Safety outcome

**FIGURE 3:**
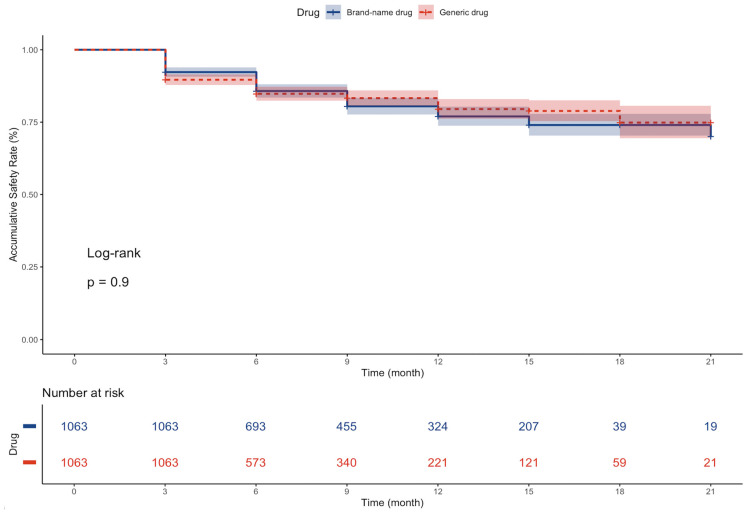
Kaplan-Meier plot for adverse events-free survival between brand-name and generic drug groups in the matched cohort. The *P*-value in survival curve was estimated using the log-rank test. Shading in the survival curve indicates 95% CIs.

In the brand-name drug group, 182 AEs were identified, compared to 161 AEs in the generic drug group. The mean AE-free survival time was 17.5 months in the brand-name drug group and 17.7 months in the generic drug group. No significant difference in total AE incidence was found between the two groups (*P* = 0.898; Figure 3). Compared with the brand-name drug group, the generic drug group did not show a statistically significant increased risk of AEs, with an adjusted HR of 1.010 (0.816–1.251) (Table 3). The most common AEs associated with pramipexole included neuropsychiatric symptoms and gastrointestinal symptoms, such as sleep disturbances, dizziness, constipation, and indigestion. Other AEs identified included orthostatic hypotension, edema, fatigue and so on. Regarding neuropsychiatric AEs, 138 cases occurred in the brand-name drug group and 124 cases in the generic drug group. No significant difference was found in the incidence of neuropsychiatric AEs between the two groups (*P* = 0.969; Supplementary Figure 3). The corresponding adjusted HR was 1.023 (0.802–1.306) (Table 3). For gastrointestinal AEs, 47 cases were observed in the brand-name drug group, and 33 cases in the generic drug group. Again, no significant difference was found between the two groups (*P* = 0.283; Supplementary Figure 4), with an adjusted HR of 0.813 (0.519–1.274). The results of the Fine-Gray model were consistent with those of primary analyses: 0.910 (0.742–1.115) for total AEs, 0.922 (0.729–1.167) for neuropsychiatric AEs, and 0.721 (0.464–1.122) for gastrointestinal AEs (Table 3).

**TABLE 3:** Comparative risk of adverse events between brand-name and generic pramipexole in the matched cohort.

	Number of events			Hazard ratio (95%CI)		
AEs	Number of matched pairs	Generic drug	Brand-name drug (ref)	Univariate cox model	Multivariate cox model	Fine-gray model
Total AEs	1,063	161	182	0.987 (0.798–1.221)	1.010 (0.816–1.251)	0.910 (0.742–1.115)
Neuropsychiatric AEs	1,063	124	138	1.005 (0.788–1.282)	1.023 (0.802–1.306)	0.922 (0.729–1.167)
Gastrointestinal AEs	1,063	33	47	0.785 (0.503–1.227)	0.813 (0.519–1.274)	0.721 (0.464–1.122)

The multivariate Cox model and Fine-Gray model were adjusted for covariates including age, gender, medical insurance type, number of disease diagnoses, levodopa equivalent dose levels at the index date, and polypharmacy (use of ≥ 5 medications simultaneously); AE, adverse event.

In the safety sensitivity analyses, 2,053 patients receiving the brand-name drug and 2,767 receiving the generic drug were initially identified. After PSM, 2,052 patients were included in each cohort, with well-balanced baseline characteristics (all *P* > 0.05; Supplementary Table 3). A total of 182 AEs were identified in the brand-name drug group, compared to 129 AEs in the generic drug group. The sensitivity analysis yielded results consistent with the primary analysis, showing no statistically significant difference in AE risks between the two groups (total AEs: *P* = 0.325; neuropsychiatric AEs: *P* = 0.513; gastrointestinal AEs: *P* = 0.247). The adjusted HRs were 0.945 (0.752–1.186) for total AEs, 0.958 (0.739–1.241) for neuropsychiatric AEs, and 0.828 (0.516–1.328) for gastrointestinal AEs.

### 3.4 Costs outcome

**FIGURE 4:**
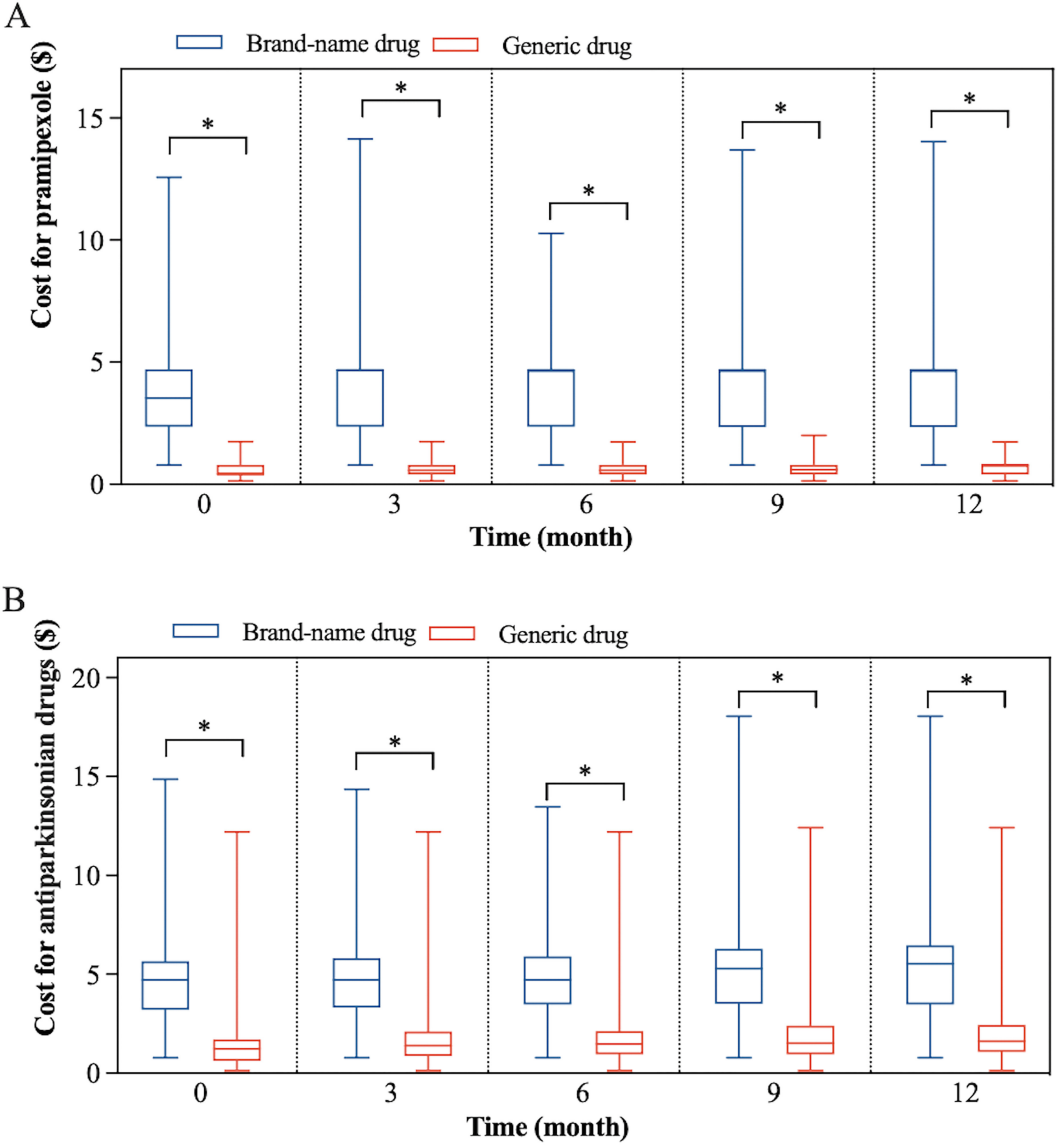
Comparisons of cost outcomes between brand-name and generic drug groups among patients with follow-up lasting more than 1 year in the matched cohort. Cost outcomes included the daily cost for pramipexole **(A)** and daily cost for antiparkinsonian drugs **(B)** at the index date and each follow-up time point. The *P*-values for pairwise comparisons were achieved with the non-parametric Mann-Whitney U tests and added for each subgroup in the graph. * refers to *P* < 0.001.

The median daily cost of pramipexole for patients with follow-up lasting more than 1 year in the matched cohort was $4.68 in the brand-name drug group and $0.58 in the generic drug group. These costs were significantly higher in the brand-name drug group compared to the generic drug group at the index date and at 3, 6, 9, and 12 months during the follow-up period (all *P* < 0.05; Figure 4A).

Similarly, the median daily cost of antiparkinsonian drugs was $4.77 in the brand-name drug group and $1.41 in the generic drug group. Significant differences in the daily cost of antiparkinsonian drugs between the two groups were observed at all these time points (all *P* < 0.05; Figure 4B).

Additionally, the average prescription cost over the 1-year follow-up period was higher in the brand-name drug group than in the generic drug group (brand-name drug: median $135.36, IQR 96.39–181.03; generic drug: median $70.65, IQR 47.01–109.74; *P* < 0.001).

## 4 Discussion

We conducted a multi-center retrospective study using real-world data to compare the effectiveness, safety, and medication costs of generic and brand-name pramipexole. The results showed no significant differences in effectiveness or safety between the two formulations. Given its lower cost, generic pramipexole can be a viable alternative to the brand-name, helping to reduce the medical burden.

Previous studies on generic pramipexole focused on sustained-release formulations, examining *in vitro* stability and handling ease (Akiyama et al., 2019) and pharmacokinetic bioequivalence in healthy subjects (Yang et al., 2023). The sustained-release formulation of pramipexole enhances patient adherence, while the immediate-release formulation allows for more convenient dose titration based on individual symptoms in clinical settings (Frampton, 2014). However, limited research has been conducted on immediate-release generic pramipexole, despite its widespread use among Chinese patients with PD. Furthermore, the clinical equivalence between generic and brand-name pramipexole remains unestablished. This is the first study to evaluate the clinical differences between generic and brand-name immediate-release pramipexole dihydrochloride tablets in PD patients. As pramipexole is the first of PD treatment drugs included in the NCDP, the findings of this study could provide valuable insights for the clinical use of generic pramipexole and promote the implementation of the NCDP policy.

Most current studies on the effectiveness and safety of drugs for PD are randomized controlled trials (RCTs), which mainly observe the changes of UPDRS scores and the occurrence of adverse reactions 2–3 months after treatment initiation (Jiang et al., 2021; Jiang et al., 2020). However, outcomes measured using validated scales such as the UPDRS are often difficult to collect in real-world settings. Therefore we adopted changes in LED as an effectiveness indicator, because LED reflects both medication intensity and, indirectly, disease severity. Longitudinal changes in LED can also provide insights into real-world fluctuations in symptom control in PD (Li et al., 2024). Considering PD, as one of the chronic diseases, is often managed or adjusted in outpatient settings (Li et al., 2024), this study utilized outpatient prescription data to ensure a sufficient sample size for analysis. This real-world study can offer broader applicability compared to RCTs and complement their findings.

Few studies have used outpatient prescription data and LED levels to analyze the effectiveness of PD treatment drugs. For example, Faddoul et al. retrospectively analyzed the changes in LED level and UPDRS scores of patients treated with pramipexole sustained-release tablets for 13 weeks at a PD treatment center in Lebanon to access drug effectiveness (Faddoul et al., 2018). To compare the effectiveness and safety of different antiparkinsonian drugs in real world, Li et al. (2024) conducted a retrospective study comparing LED levels and the occurrence of AEs. This study, with a larger sample size, employed PSM to balance baseline PD severity and medication strength by equalizing enrollment LED levels. We found no significant differences in the change of LED levels over time between generic and brand-name pramipexole. Sensitivity analyses confirmed the robustness of the findings.

For safety assessment, this study identified AEs with a causality level of “possible” or higher, based on patients’ new diagnosis and drug use, along with time sequence (Butler et al., 2022; Savage et al., 2023). The most common AEs were neuropsychiatric symptoms and gastrointestinal symptoms, consistent with the drug’s instructions and related studies (CSPDMD and PDMD-CMDA, 2020; Chen et al., 2020). No significant differences were observed in total AEs, neuropsychiatric symptoms, or gastrointestinal symptoms between groups. To evaluate real-world, longer-term safety, the primary analysis focused on patients with at least one follow-up visit after enrollment. Patients without follow-up records were excluded from the main safety population, as their absence could stem from multiple factors (e.g., care transition, intolerance, treatment failure, or financial reasons). Nonetheless, a sensitivity analysis incorporating these patients demonstrated that the findings remained robust.

Pramipexole was once among the most expensive PD medications. As reported by Yi et al. (2022) the highest average cost per visit or admission episode for PD patients was incurred by pramipexole from 2016 to 2018. This study highlighted the economic benefits of selected generic pramipexole in NCDP. The cost of a single tablet of generic pramipexole dihydrochloride tablets is about one-tenth that of the brand-name drug, and study has shown that patients using generic pramipexole experience a reduction of up to 80% in daily medication costs compared to the brand-name drug. Both the daily cost of antiparkinsonian drugs and the total drug expenditure of patients have also decreased significantly. The NCDP policy has effectively lowered procurement prices, accelerated generic substitution, saved expenditure, and reduced patient out-of-pocket costs (Jiang et al., 2025; Wang et al., 2023; Zhao and Wu, 2023). It also improved treatment coverage and equity (Liu et al., 2025). No previous studies have analyzed the overall financial burden on patients using generic antiparkinsonian drugs. Our study demonstrates the cost advantage of generics with comparable efficacy and safety. Future studies should collect detailed data on expenses including PD-related clinical visits and hospitalizations. This would allow for a complete assessment of healthcare resource utilization and, ultimately, an evaluation of the cost-effectiveness of the generic drug.

Some limitations should be noted. First, following the implementation of NCDP, only the generic version of pramipexole was available in the research hospital’s formulary, meaning the two groups were treated in different time periods. To minimize the potential impact of this factor, strict population selection criteria and consideration of time in statistics have been adopted in this study. Nevertheless, the design involving different study periods inevitably introduces potential influences such as the coronavirus disease 2019 (COVID-19) pandemic. Although PSM balanced observed covariates, unmeasured confounding related to the pandemic cannot be completely ruled out. Second, due to limitations in prescription records, direct data on the drug’s effectiveness and safety were unavailable. Indirect indicators may bias results. The inclusion criterion of requiring at least four follow-up records may have led to an overestimation of pramipexole’s efficacy. However, as it affected both groups equally, the comparative findings between the groups are still interpretable. AE incidence could also be influenced by prescribing practices. Additionally, since this was a retrospective cohort study, discrepancies may exist between prescribed and actual adherence. Lastly, while this study was multicenter and included a larger sample size compared to previous studies, the unavailability of patient records from other medical centers could also introduce bias into the results. Data from an even larger cohort would be valuable, and the use of target trial emulation could further enhance the rigor and reproducibility of the findings (Wang et al., 2026). Prospective studies should be conducted to validate the present findings.

## 5 Conclusion

This study demonstrated that the effectiveness and safety of generic pramipexole are comparable to those of the brand-name drug. The use of generic pramipexole offers a significant reduction in medication costs for PD patients. It can be used as an effective alternative to the brand-name drug. Future prospective studies with larger sample sizes should be carried out to further provide evidence for the rational use of generic drugs in clinical practice.

## Data Availability

The original contributions presented in this study are included in this article/Supplementary material, further inquiries can be directed to the corresponding authors.
