# Supplementary material for: Generic vs. brand-name pramipexole: effectiveness, safety, and cost comparison in a multicenter retrospective cohort study
Source: Front Aging Neurosci. 2026 Jun 29;18:1873459. doi: 10.3389/fnagi.2026.1873459 (PMC13357990; doi:10.3389/fnagi.2026.1873459)
Supplement: Supplementary file 1 [file Data_Sheet_1.DOCX]

Generic vs. Brand-Name Pramipexole: Effectiveness, Safety, and Cost Comparison in a Multicenter Retrospective Cohort Study

Supplementary Material

## Supplementary Figures


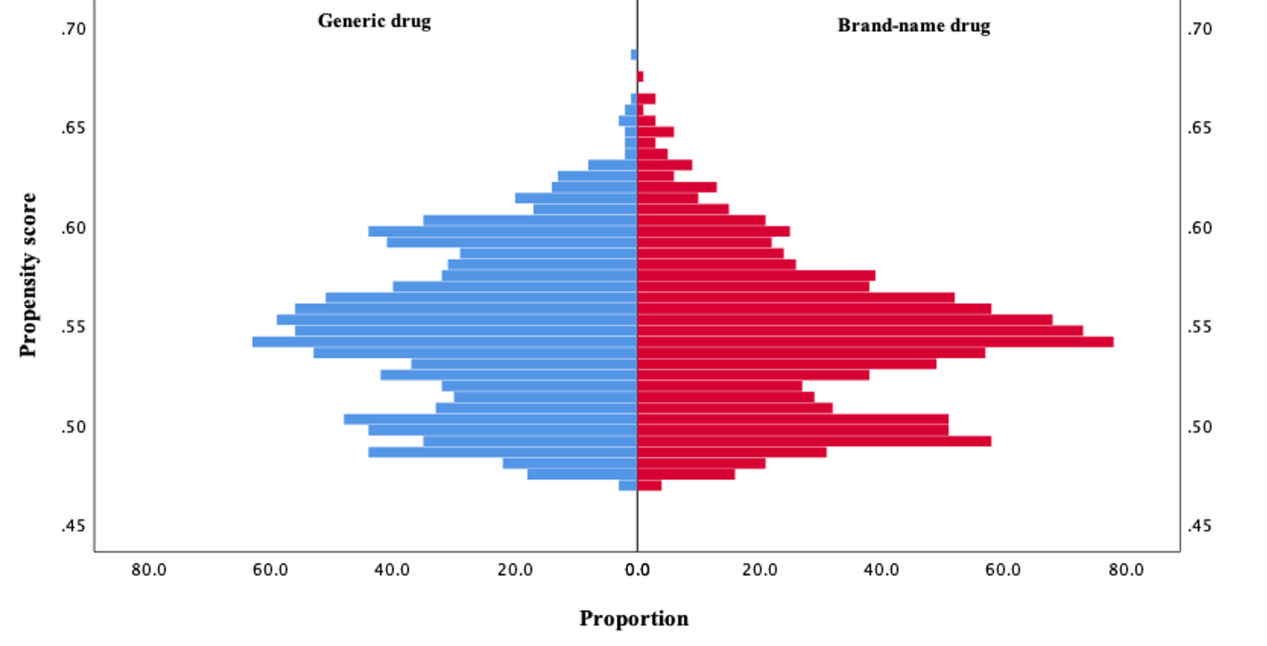


**Supplementary Figure 1.** Distribution of propensity scores after matching between brand-name and generic pramipexole groups


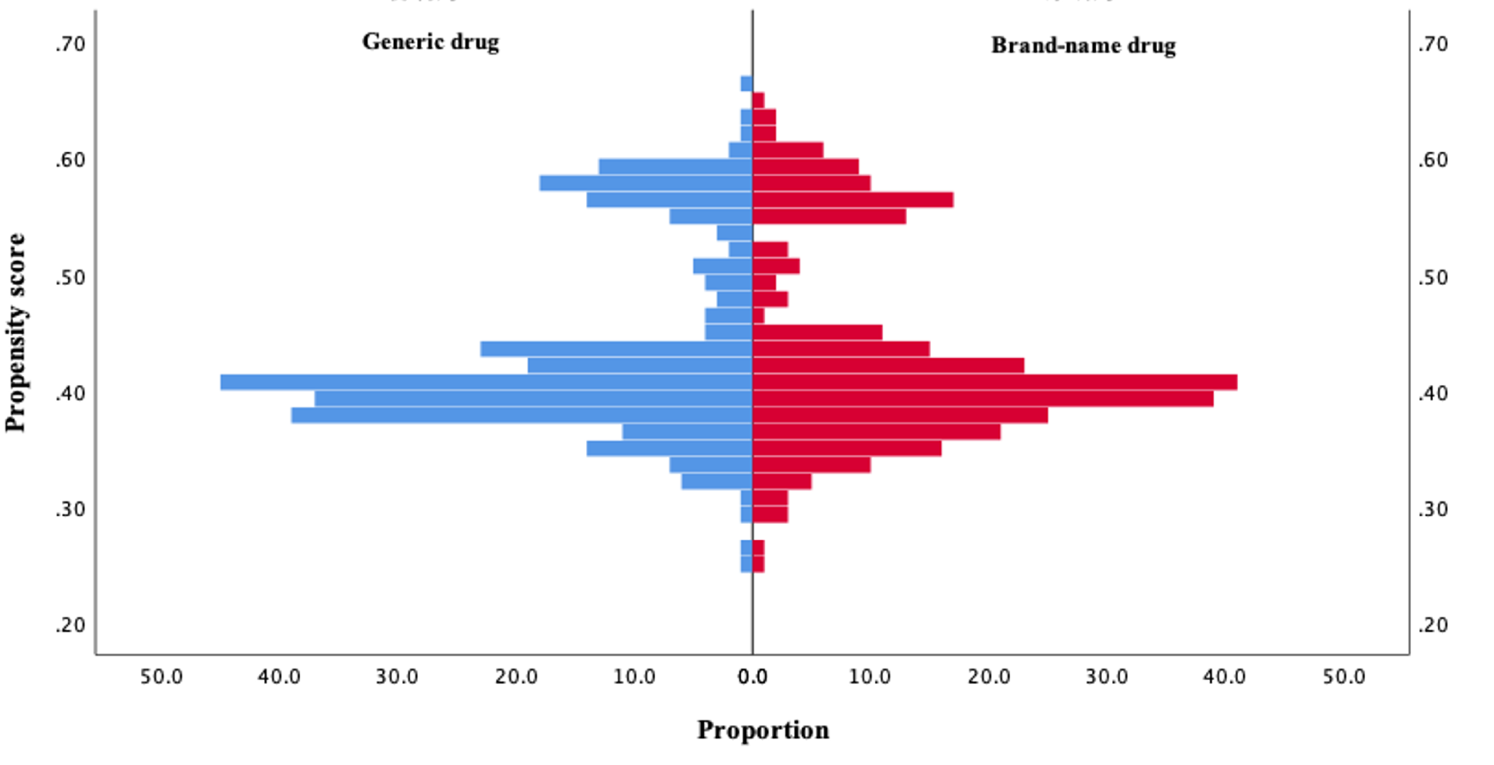


**Supplementary Figure 2.** Distribution of propensity scores after matching between patients with follow-up lasting more than one year using brand-name and generic pramipexole


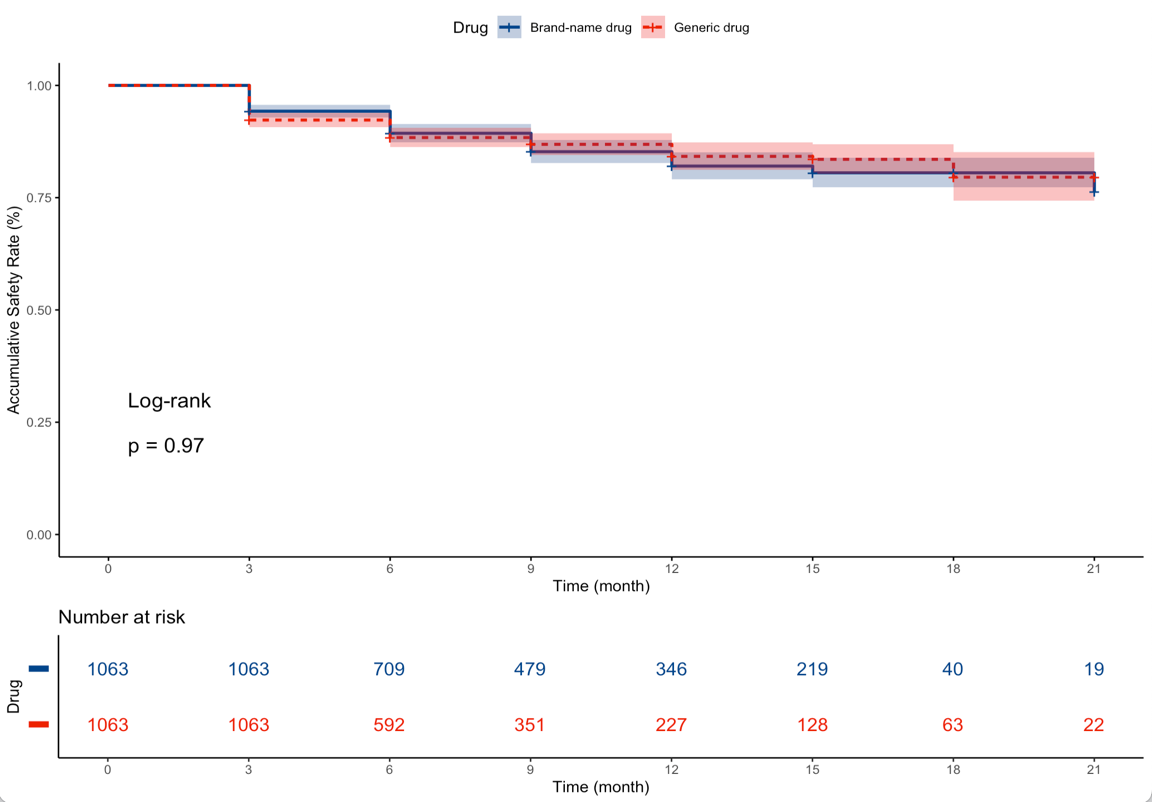


**Supplementary Figure 3.** Kaplan-Meier plot for neuropsychiatric adverse events-free survival between brand-name and generic drug groups in the matched cohort. The P value in survival curve was estimated using the log-rank test. Shading in the survival curve indicates 95% CIs.


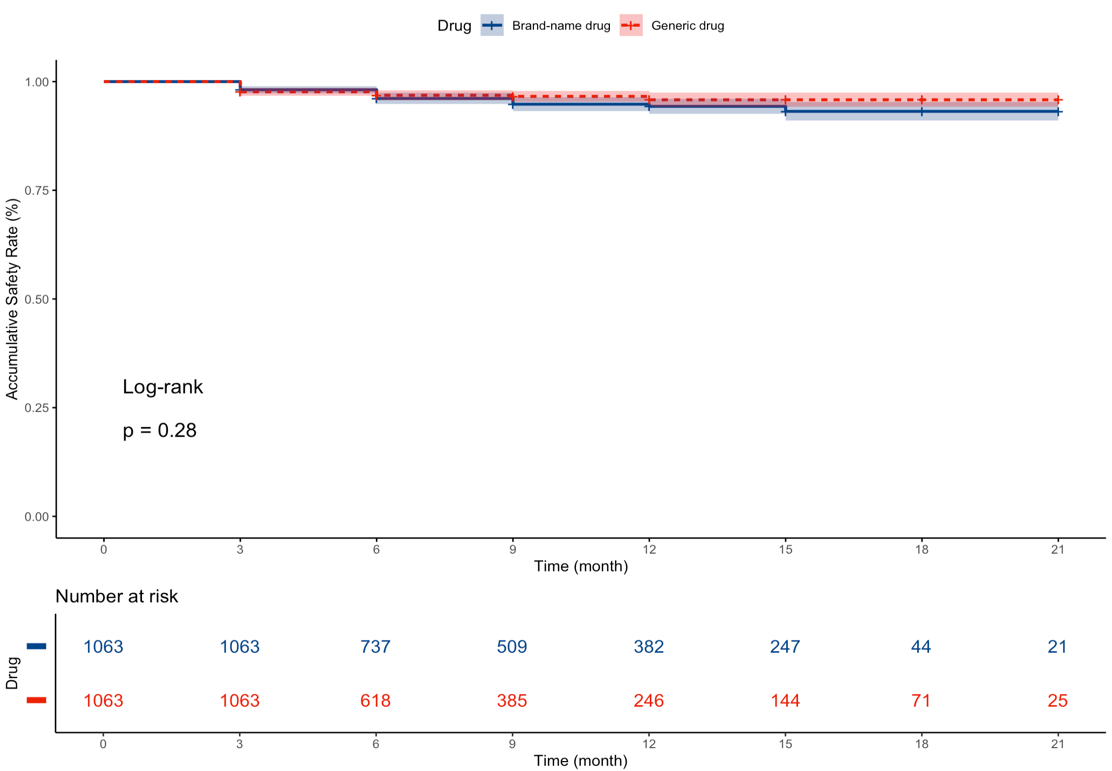


**Supplementary Figure 4.** Kaplan-Meier plot for gastrointestinal adverse events-free survival between brand-name and generic drug groups in the matched cohort. The P value in survival curve was estimated using the log-rank test. Shading in the survival curve indicates 95% CIs.

## Supplementary Tables

**Supplementary Table 1.** Baseline characteristics of patients with at least five follow-up records using brand-name and generic pramipexole

| Characteristics | Unadjusted | | | Propensity score-matched | | |
| --- | --- | --- | --- | --- | --- | --- |
|  | Brand-name drug (n=262) | Generic drug  (n=182) | *P* value | Brand-name drug (n=160) | Generic drug  (n=160) | *P* value |
| Gender, n (%) |  |  | 0.952 |  |  | 0.370 |
| Male | 126 (48.1) | 87 (47.8) |  | 72 (45.0) | 80 (50.0) |  |
| Female | 136 (51.9) | 95 (52.2) |  | 88 (55.0) | 80 (50.0) |  |
| Age, years; mean ± SD | 66.9 ± 9.93 | 67.2 ± 10.02 | 0.768 | 66.4 ± 9.62 | 67.0 ± 10.02 | 0.602 |
| Medical insurance type, n (%) |  |  | ＜0.001 |  |  | 0.889 |
| With health insurance | 229 (87.4) | 130 (71.4) |  | 128 (80.0) | 127 (79.4) |  |
| No health insurance | 33 (12.6) | 52 (28.6) |  | 32 (20.0) | 33 (20.6) |  |
| No. of disease diagnoses, median (IQR) | 1 (1-3) | 1 (1-2) | 0.050 | 1 (1-3) | 1 (1-2) | 0.313 |
| LED levels at the index date, mg; median (IQR) | 550.0 (150.0-850.0) | 637.5 (150.0-825.0) | 0.998 | 712.5 (150.0-939.1) | 662.5 (150.0-850.0) | 0.121 |
| Patients on polypharmacy, n (%) | 32 (12.2) | 24 (13.2) | 0.761 | 19 (11.9) | 18 (11.3) | 0.861 |

Polypharmacy refers to using of five or more medications simultaneously

*SD* standard deviation, *IQR* interquartile range, *LED* levodopa equivalent dose

**Supplementary Table 2.** Comparisons of LED levels at each follow-up time point between brand-name and generic drug groups among patients with at least five follow-up records in the matched cohort

| LED, mg; median (IQR) | Brand-name drug (n=160) | Generic drug (n=160) | *P* value |
| --- | --- | --- | --- |
| 3 months | 750.0 (150.0-950.0) | 675.0 (184.4-950.0) | 0.514 |
| 6 months | 750.0 (215.6-950.0) | 675.0 (250.0-950.0) | 0.328 |
| 9 months | 750.0 (165.6-1050.0) | 675.0 (256.3-975.0) | 0.333 |
| 12 months | 750.0 (225.0-1050.0) | 750.0 (387.5-1025.0) | 0.789 |

*IQR* interquartile range, *LED* levodopa equivalent dose

**Supplementary Table 3.** Baseline characteristics of patients in the sensitivity analyses for safety

| Characteristics | Unadjusted | | | Propensity score-matched | | |
| --- | --- | --- | --- | --- | --- | --- |
|  | Brand-name drug (n=2053) | Generic drug  (n=2767) | *P* value | Brand-name drug (n=2052) | Generic drug  (n=2052) | *P* value |
| Gender, n (%) |  |  | 0.481 |  |  | 0.755 |
| Male | 1042 (50.8) | 1376 (50.3) |  | 1042 (50.8) | 1032 (50.3) |  |
| Female | 1011 (49.2) | 1391 (49.7) |  | 1010 (49.2) | 1020 (49.7) |  |
| Age, years; median (IQR) | 66.0 (59.0-73.0) | 67.0 (59.0-73.0) | 0.022 | 66.0 (59.0-73.0) | 66.1 (59.0-73.0) | 0.220 |
| Medical insurance type, n (%) |  |  | 0.539 |  |  | 0.595 |
| With health insurance | 1108 (54.0) | 1518 (54.9) |  | 1108 (54.0) | 1091 (53.2) |  |
| No health insurance | 945 (46.0) | 1249 (45.1) |  | 944 (46.0) | 961 (46.8) |  |
| No. of disease diagnoses, median (IQR) | 1 (1-2) | 1 (1-2) | 0.012 | 1 (1-2) | 1 (1-2) | 0.212 |
| LED levels at the index date, mg; median (IQR) | 325.0 (75.0-750.0) | 425.0 (100.0-725.0) | <0.001 | 331.3 (75.0-750.0) | 375.0 (75.0-675.0) | 0.240 |
| Patients on polypharmacy, n (%) | 218 (10.6) | 325 (11.7) | 0.221 | 218 (10.6) | 226 (11.0) | 0.688 |

Polypharmacy refers to using of five or more medications simultaneously

*SD* standard deviation, *IQR* interquartile range, *LED* levodopa equivalent dose
